# Deciphering RNA splicing logic with interpretable machine learning

**DOI:** 10.1073/pnas.2221165120

**Published:** 2023-10-05

**Authors:** Susan E. Liao, Mukund Sudarshan, Oded Regev

**Affiliations:** ^a^Department of Computer Science, Courant Institute of Mathematical Sciences, New York University, New York, NY 10012

**Keywords:** RNA splicing, interpretable machine learning, artificial intelligence

## Abstract

Machine learning methods, particularly neural networks trained on large datasets, are transforming how scientists approach scientific discovery and experimental design. However, current state-of-the-art neural networks are limited by their uninterpretability: Despite their excellent accuracy, they cannot describe how they arrived at their predictions. Here, using an “interpretable-by-design” approach, we present a neural network model that provides insights into RNA splicing, a fundamental process in the transfer of genomic information into functional biochemical products. Although we designed our model to emphasize interpretability, its predictive accuracy is on par with state-of-the-art models. To demonstrate the model’s interpretability, we introduce a visualization that, for any given exon, allows us to trace and quantify the entire decision process from input sequence to output splicing prediction. Importantly, the model revealed uncharacterized components of the splicing logic, which we experimentally validated. This study highlights how interpretable machine learning can advance scientific discovery.

## Significance

Machine learning approaches are increasingly applied to advancing discovery in the biological sciences. However, despite achieving predictive accuracy, many machine learning models cannot explain how they achieve their predictive success. Here, we demonstrate that bespoke data generation coupled with model design that infuses foundational biological knowledge enables an “interpretable-by-design” approach that advances our understanding of RNA splicing. Our model not only accurately predicts the quantitative splicing outcomes but also explains how specific combinations of RNA features dictate splicing outcomes. We validate the network predictions and interpretations through additional data generation and experimental validation. These results demonstrate that “interpretable-by-design” machine learning represents a powerful approach to harnessing the potential of machine learning toward advancing our understanding of biological processes.

Machine learning algorithms, in particular neural networks, capture complex quantitative relationships between input and output. However, as neural networks are typically black boxes, it is difficult to extract post hoc insights into how they achieve their predictive success. Furthermore, they easily capture artifacts or biases in the training data, often fail to generalize beyond the datasets used for training and testing, and do not lead to insights into the underlying processes (1).

In recent years, neural networks have been used to tackle challenging biological questions. One outstanding question in genomics is understanding the regulatory logic of RNA splicing, which plays a critical role in the fundamental transfer of information from DNA to functional RNA and protein products. Splicing removes introns and ligates exons together to form mature RNA transcripts. While some canonical sequence features are necessary for exon definition (splice sites delimiting exons and branch points used during intron removal), exon definition is also facilitated by exon sequence (2, 3). Despite recent success using neural networks to predict splicing outcomes (4, 5), understanding how exon sequence dictates inclusion or skipping remains an open challenge. The challenge is further underscored by the sensitivity of splicing logic, where almost all single-nucleotide changes along an exon can lead to dramatic changes in splicing outcomes (6, 7).

To enable scientific progress, machine learning models should not only accurately predict outcomes but also describe how they arrive at their predictions. Here, we demonstrate that an “interpretable-by-design” model achieves predictive accuracy without sacrificing interpretability, captures a unifying decision-making logic, and reveals previously uncharacterized splicing features.

## Results

### Generating a Synthetic Dataset for Interpretable Machine Learning.

As neural network performance and interpretability are inextricable from the data it is trained on, we began by generating a large, high-quality synthetic splicing dataset. The use of synthetic datasets offers several advantages over genomic data used in previous work. First, genomic datasets are limited by the number of exons in the genome. In contrast, synthetic assays can dramatically increase the number of data points by orders of magnitude (8, 9). Second, genomic exons are flanked by varying sequences (splice sites, introns, promoters) that also participate in splicing decisions (10), greatly complicating attempts at interpretability. In contrast, synthetic datasets fix all but one variable region, allowing one to focus on the region of interest. Third, genomic exons contain overlapping RNA codes (e.g., protein-coding sequences). In contrast, sequences in synthetic datasets are devoid ofoverlapping codes by design. In summary, from both a quantity and quality perspective, synthetic datasets provide crucial advantages for machine learning over genomic datasets.

**Fig. 1.**
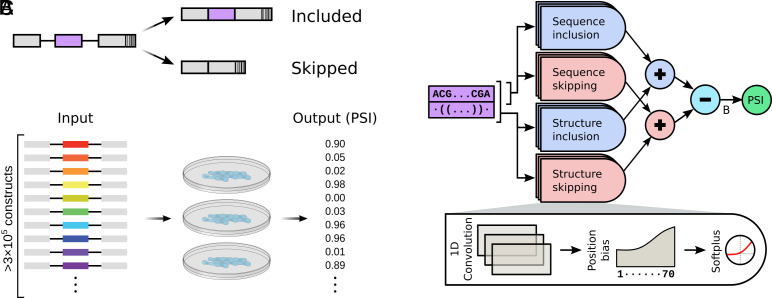
Data generation and interpretable-by-design machine learning model. (*A*) All reporters in the assay share the same three-exon design and differ only in their middle exon, which contains a random 70-nucleotide-long sequence. Depending on its sequence, an exon might be included, skipped, or a probabilistic mix of the two. Each reporter includes a unique barcode at the end of the third exon so that exon identity can be inferred in exon-skipping products. (*B*) The assay includes over $3\times 10^{5}$ different reporters. The reporters were transfected into HeLa cells in a pooled fashion in three biological replicates. High-throughput sequencing then provides a “percent spliced in” (PSI) value to each reporter. (*C*) The machine learning model consists of both short convolution filters (applied to exon sequence only) and long convolution filters (applied to both exon sequence and predicted structure). The output of these filters (strength) can depend on the position along the exon. Half of the filters are designated as inclusion filters, and the rest are skipping filters. Predicted PSI is computed from the difference between the total strength of inclusion filters and the total strength of skipping filters, after adding an initial basal strength (B).

The synthetic dataset we generated includes hundreds of thousands of input–output data points. Each data point is a different random 70-nucleotide exon sequence, paired with a measured percent spliced in (PSI) output, which is a number between 0 (always skipped) and 1 (always included) (Fig. 1*A*). The dataset is generated by a massively parallel reporter assay that allows for PSI quantification for hundreds of thousands of unique sequences in a single biological experiment (Fig. 1*B*). Splicing outcomes for the parallel reporter assay were measured after transfection into human HeLa cells using high-throughput sequencing. We confirmed that reporters are evenly represented in the reporter assay (*SI Appendix*, Fig. S1*A*). The vast majority of splicing products corresponded to exon inclusion or exon-skipping products (*SI Appendix*, Fig. S1*B*), and we filtered our data to exclude spurious splicing products. PSI values are calculated as the number of inclusion reads divided by the total number of inclusion and skipping reads. Three biological replicates of the assay showed excellent agreement (*SI Appendix*, Fig. S1*C*), and their sequencing results were combined for all downstream analyses. High-throughput sequencing measurements were consistent with semiquantitative measurements of individual reporters (*SI Appendix*, Fig. S1*D*).

### An Interpretable-by-Design Model Accurately Predicts Splicing Outcomes.

We first compared the predictive accuracy of three off-the-shelf machine learning algorithms on our dataset: a k-mer splicing scoring algorithm (8) (*SI Appendix*, Fig. S2*A*), a gated recurrent unit neural network (11) (*SI Appendix*, Fig. S2*B*), and a transformer neural network (12) (*SI Appendix*, Fig. S2*C*). We found that the two neural networks outperformed the k-mer scoring algorithm. This gap in predictive accuracy suggests that the more complex neural networks capture additional features affecting splicing outcomes. However, as these models are not interpretable, we were unable to pinpoint which specific features contribute to the improved predictions.

We therefore designed a neural network model with the explicit goal of being interpretable (13). The predictive accuracy of our interpretable-by-design model is comparable to that of the two state-of-the-art neural networks (*SI Appendix*, Fig. S2*D*). This suggests that interpretability need not come at the expense of accuracy.

In addition to our own dataset, the model accurately predicts splicing outcomes from other splicing datasets (7, 8, 14–17) (*SI Appendix*, Table S1 and Fig. S3). Importantly, unlike our random exons, these datasets were modeled on specific genomic exons, with each dataset differing in splice sites, introns, and flanking exons. Furthermore, these datasets were generated in different immortalized cell lines. Encouragingly, despite these dramatic differences in RNA architecture and cell types, our model performed well on these datasets, suggesting that our model generalizes and captures critical aspects of splicing regulatory logic.

### Model Architecture Reveals Unifying Decision-Making Process.

Our interpretable-by-design model incorporates domain knowledge throughout its architecture (Fig. 1*C*). Specifically, we reasoned that short six-nucleotide sequence filters would capture motifs previously demonstrated to play an important role in splicing decisions (18, 19). We therefore introduced one-dimensional convolutional filters applied to the input RNA sequence. Next, since RNA secondary structure was previously implicated in splicing outcomes (16, 20), we also provided the network with predicted structure (21). We then introduced longer (30-nucleotide) one-dimensional convolutional filters to the structure-augmented sequence. Crucially, while we fixed filter lengths using minimal domain knowledge, we did not explicitly specify sequences and structures, allowing the network flexibility to learn filters in an unbiased manner. Furthermore, our model explicitly quantifies the strength (in network-defined arbitrary units) of each activated filter to the inclusion or skipping decision. Importantly, we allowed the strength of any filter to vary along the length of an exon, providing the network the flexibility to capture position-dependent effects of RNA features on splicing outcomes.

To arrive at its output, the network computes the difference in the sum total of exon inclusion strengths and exon skipping strengths (Δ strength), which is then converted to predicted PSI. The greater the magnitude of this difference, the closer the PSI is to 0 (difference≪0) or 1 (difference≫0). This additive combinatorial behavior is consistent with the previous literature (8, 22).

### Model Extends Understanding of Splicing Regulatory Logic.

Even though our model was trained on a synthetic dataset, it recapitulates and extends domain knowledge from previous genomic and biochemical studies.

**Fig. 2.**
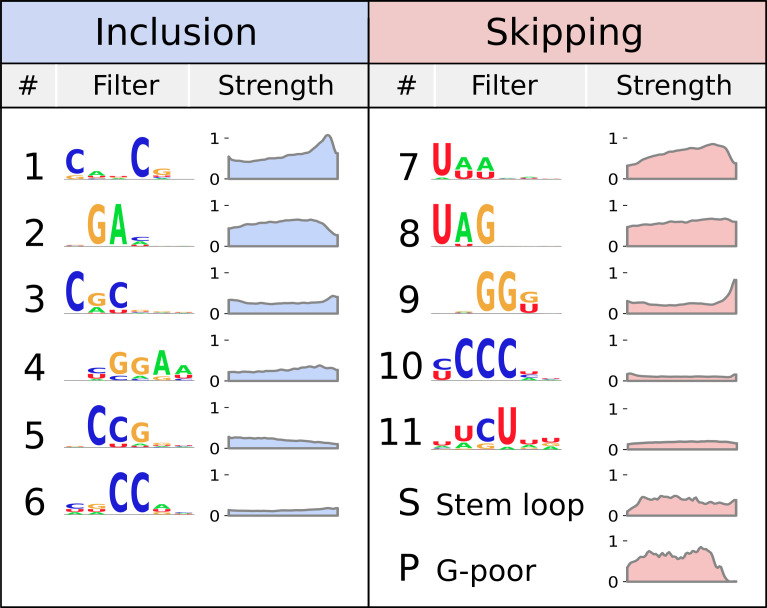
Model expands on known splicing logic. Splicing features detected by the model’s filters, represented by their sequence logo (25). Filters either contribute to inclusion (blue) or skipping (red). Plots show the average strength in our dataset of each filter as a function of position along the exon. The model also identified short stem loops and long G-poor stretches as contributing to exon skipping.

Many filters in the model match binding motifs of RNA-binding proteins implicated in splicing regulation (splicing factors) (23, 24) (Fig. 2 and *SI Appendix*, Fig. S4). Consistent with previous studies, network inclusion filters match binding sites for SR proteins known to promote exon inclusion (26, 27), whereas network skipping filters match binding sites for hnRNP proteins known to promote exon skipping (28).

However, while the directionality of these RNA features toward splicing was established, their magnitude was not clear. Importantly, the model addresses this issue by assigning a quantitative strength to each filter. Moreover, some filters exhibit striking position-dependent strengths, suggesting that the position of an RNA feature along an exon affects its strength. This is consistent with previous experimental reports demonstrating position-dependent effects of RNA sequences within exons (8).

Surprisingly, our network accurately predicted splicing outcomes using a concise list of filters (Fig. 2). This contrasts with previous studies suggesting that splicing outcomes result from the combinatorics of hundreds of unique RNA features (8, 29, 30).

**Fig. 3.**
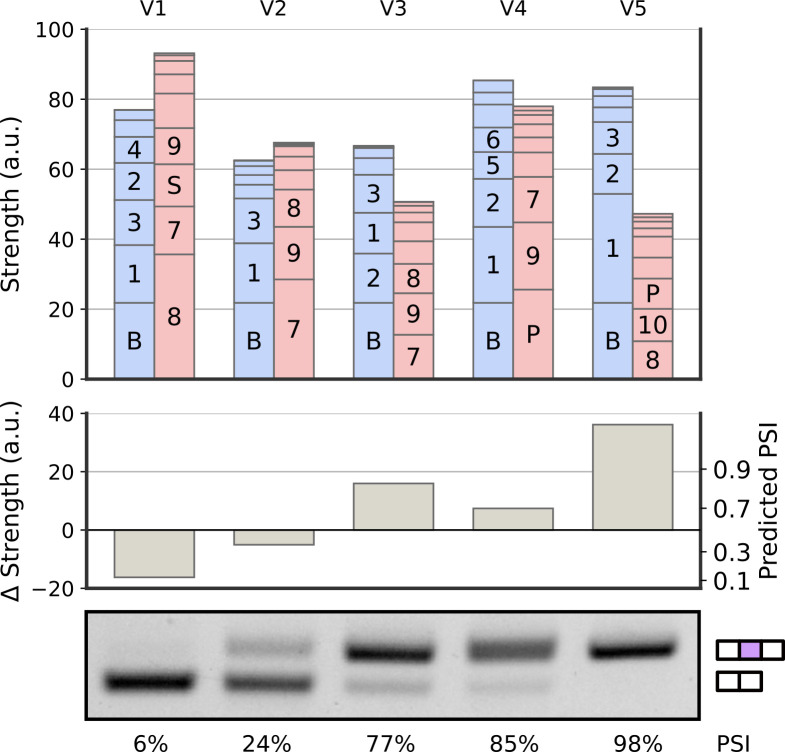
Model predictions can be interpreted using balance plots. Balance plots used to visualize the logic leading to PSI prediction for five randomly picked exons (V1–V5). Bar plot showing the total strength contributed by each filter (*Top*). Bars are labeled by filter numbers from Fig. 2. The bar labeled B represents a fixed initial basal strength. Labels are not shown for smaller bars. The difference between total inclusion and total skipping strengths (Δ strength) leads to predicted PSI (*Middle*). PSI as measured by semiquantitative RT-PCR matches the machine learning predictions (*Bottom*).

Using the local interpretability of our model, we introduce a visualization (balance plot) that enables explicit examination and quantification of how multiple RNA features lead to splicing outcomes for any given exon from our dataset (Fig. 3, *SI Appendix*, Fig. S6) and other datasets (*SI Appendix*, Fig. S5). For a given exon, the total strengths of activated filters are represented as bars of the appropriate height. Total inclusion strength (blue) and skipping strength (red) are then visible as the height of the stacked bars. The Δ strength is represented by the difference in heights between the stacked inclusion and skipping filters. These visualizations provide an intuitive tool to understand the contributions of individual sequence and structure features leading to each exon’s predicted PSI. They emphasize that splicing logic results from contributions of many RNA features along the exon and that a single nucleotide can be part of multiple overlapping filters (6, 8).

### Identification and Validation of Uncharacterized Splicing Features.

Next, we asked whether our interpretable-by-design model could identify uncharacterized splicing features. While most network filters were consistent with previously described splicing features, two uncharacterized long skipping filters with strong influence on splicing predictions stood out (Fig. 2). We confirmed that these filters were robustly identified across multiple initialization seeds and training/testing splits, suggesting that they are not training artifacts. We then turned our attention to characterizing and experimentally validating these features.

**Fig. 4.**
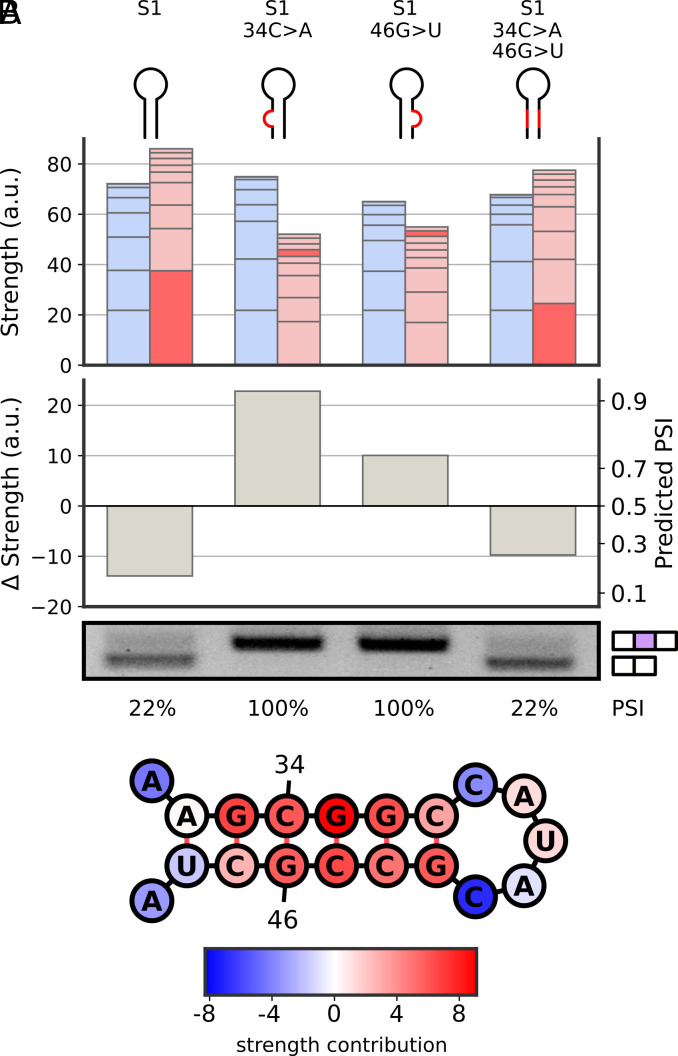
Validation of the stem loop feature. (*A*) The machine learning model identifies a stem loop in an exon (S1) as having a strong skipping strength (dark red bar; *Top*), leading to near-complete skipping prediction (*Middle*). Single-nucleotide mutations disrupting a downstream or upstream stem base pair are predicted to significantly reduce the skipping strength and restore exon inclusion. Finally, including both single-nucleotide mutations is predicted to restore the stem loop skipping strength and lead to skipping. RT-PCR validation (*Bottom*) confirms the machine learning predictions. (*B*) The stem loop identified in S1, with the individual contributions to its strength by each nucleotide.

Examining the first uncharacterized filter revealed that it identifies stem loop structures with short, GC-rich, 5-7 nucleotide double-stranded regions (Fig. 4). Next, we experimentally validated that these stem loops contribute to exon skipping and are not artifacts. We introduced mutations that disrupt double-stranded base pairing in an exon with such a stem loop. First, we introduced single-nucleotide mutations predicted to abolish the stem by disrupting base pairing. Notably, these mutations were designed to minimize disruptions of other filters, ensuring that prediction differences are mainly due to altered secondary structure, and not due to the introduction or disruption of other sequence features. In addition to two such mutations, we also introduced both compensatory mutations together, restoring the original stem loop structure (31). We measured splicing outcomes for all four individual reporters (original, upstream mutation, downstream mutation, and double mutations) and observed that splicing outcomes matched our predictions (Fig. 4). Namely, PSI increased dramatically in both single-nucleotide mutants, in agreement with the predicted decrease in filter strength. Furthermore, when both compensatory mutations are present and structure is restored, measured PSI was comparable to that of the original exon. We applied the same experimental validation scheme to four other stem loop-containing exons. In all cases, stem-disrupting single mutations increased exon inclusion, and structure-restoring double compensatory mutations had minimal effects (*SI Appendix*, Fig. S7). Together, these experiments demonstrate that model-identified stem loops, rather than sequence, contribute to exon skipping.

**Fig. 5.**
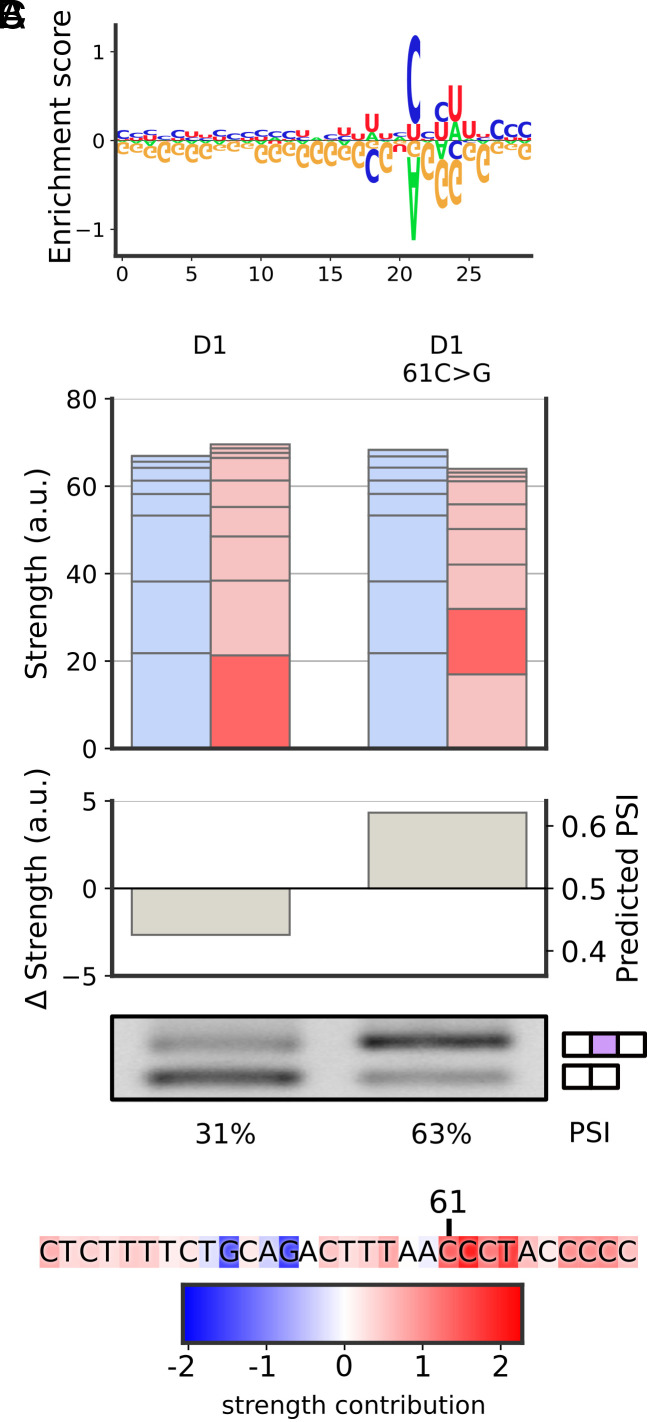
Validation of the G-poor feature. (*A*) The G-poor filter, represented by its enrichment-depletion logo (32). (*B*) The machine learning model identifies a G-poor stretch in an exon (D1) as having a strong skipping strength (dark red bar, *Top*), leading to skipping prediction (*Middle*). A single-nucleotide C>G mutation is predicted to disrupt the G-poor stretch and restore exon inclusion (*Right* bars). RT-PCR validation (*Bottom*) confirms the machine learning predictions. (*C*) The G-poor stretch identified in D1, with the individual contributions to its strength by each nucleotide.

In contrast, examining the second uncharacterized filter did not reveal any secondary structure preference. Instead, the filter exhibited a preference for long guanine-depleted (G-poor) sequences (Fig. 5*A*). To validate that guanine depletion underlies filter behavior, we selected an exon with a G-poor sequence and introduced a single C>G mutation. As before, we ensured that the predicted strengths of other filters are only minimally disrupted (Fig. 5*B*). Strikingly, this single mutation led to a marked increase in PSI. We applied the same validation scheme to three other exons with G-poor sequences; in every instance, a single C>G mutation increased exon inclusion (*SI Appendix*, Fig. S8). To the best of our knowledge, a long G-poor sequence has not been described in the literature.

Collectively, these experiments confirm that stem loops and G-poor sequences identified by the model reflect bona fide splicing features.

## Discussion

In this study, we demonstrate that an interpretable-by-design model advanced scientific discovery. Our model accurately predicts splicing outcomes on both our assay and on previously published assays, demonstrating that interpretability need not come at the expense of accuracy or generalizability. Model interpretability enabled a systematic understanding of RNA splicing logic, including the identification of two candidate exon-skipping features which were subsequently experimentally validated. The model’s ability to quantify contributions of specific features to splicing outcomes for individual exons has considerable potential for a range of medical and biotechnology applications, including genome- or RNA-editing of target exons to correct splicing behavior or guiding rational design of RNA-based therapeutics like antisense oligonucleotides (33).

In addition, model-identified features hint at novel biochemical mechanisms that warrant further study. For example, the fact that splicing decisions are modeled well by an additive quantity (Δ strength) supports a biochemical mechanism involving the nuclear spatial organization of SR and hnRNP proteins (34). Furthermore, our model identified two unusual exon-skipping features. These features may be recognized by an uncharacterized RNA-binding protein or complex. Alternatively, the introduction of highly structured or unstructured regions may change the physical distance between splice sites, enhancing exon skipping. These open questions further underscore how interpretable-by-design models can advance scientific discovery by aiding hypothesis generation.

Our model performs well on synthetic datasets from immortalized cell lines, yet further work is needed to capture the dynamics of developmentally regulated splicing logic (35–37). Importantly, splicing outcomes change depending on the expression level of cell type–specific RNA-binding proteins (38). These questions can be addressed by generation of additional synthetic splicing datasets in developmentally relevant cell types paired with interpretable-by-design models that capture cell type–specific regulatory features.

Beyond the context of splicing, the interpretable-by-design framework can be used to decipher the multiple, complex, and overlapping codes that dictate biomolecular processing. Importantly, many rich synthetic datasets that address RNA untranslated 5’ (39) and 3’ (40) region regulation, methylation (41), and small RNA biogenesis (42) have already been generated. We expect that additional data generation efforts paired with the interpretable-by-design framework will stimulate advances in understanding biological codes more broadly.

## Materials and Methods

### Reporter Assay Design and Cloning.

The splicing reporter is based on a three-exon beta globin minigene (43) under the control of a truncated mammalian CAG promoter. The massively parallel splicing assay allows for high-throughput characterization of exon variants on splicing outcomes (44) using Gibson assembly and ligation cloning. The assay replaces the middle beta globin exon with 70-nt random sequences flanked by weak splice sites [MaxEnt scores (45): 3’ss 9.41, 5’ss 5.06]. Each 70-nt exon is coupled with a 20-nt barcode downstream of the third exon, allowing for identification of middle exon identity in exon-skipping products. Random exons and barcodes were synthesized separately as degenerate single-stranded oligonucleotides (IDT) and were joined using an anneal-extend procedure as follows. In a 100uL reaction (Phusion^®^Hot Start Flex 2X Master Mix, NEB), 200nM exon and barcode oligonucleotides were denatured at 98°C for 10 min, cooled slowly to 60°C (0.1°C/s), annealed at 60°C for 5 min, and extended at 72°C for 60 min. Single-stranded products were removed from pooled double-stranded exon–barcode using a silica column purification according to the manufacturer’s specifications (ZymoPURE Plasmid Miniprep Kit). Pooled exon–barcode products were cloned into a backbone digested with BsmBI and XbaI and expanded using electrocompetent bacterial cells (ElectroMAX™DH10B Cells, ThermoFisher) on large solid agar Bioassay plates (Nunc™Square BioAssay Dishes, ThermoFisher). After resuspending pooled bacteria in 1X PBS, DNA was recovered using silica column purification (ZymoPURE II Plasmid Maxiprep Kit, Zymo Research) following the manufacturer’s specifications. The resulting pooled library (Lib1) includes the truncated CAG promoter, followed by the first minigene exon and intron, and the exon–barcode insertion. High-throughput amplicon sequencing of Lib1 was used to match exon–barcodes pairs. To generate the final splicing reporter assay (Lib2), a fixed sequence, containing the second intron and third exon, was introduced to separate exons from their barcodes. Lib1 was digested with Esp3I (NEB) to introduce overhangs between the exons and barcodes; the digested product was gel-purified to facilitate downstream cloning (Zymoclean Gel DNA Recovery Kits). A segment containing the second intron and third exon was ligated into the digested Lib1 product (NEB Quick Ligation). Lib2 library was expanded using electrocompetent bacteria cells resulting in about 10 times as many colonies as Lib1 to ensure even representation across reporters and recovered using silica column purification as described for Lib1. DNA was quantified using a spectrophotometer (NanoDrop™One^C^, Fisher Scientific).

### Individual Reporter Cloning.

To validate consequences of point mutations on splicing outcomes, individual exons were synthesized as two single-stranded oligonucleotides (IDT DNA Technologies) and joined using an anneal-extend procedure. Briefly, 200 nM of each oligonucleotide were joined in a 100 μL reaction with 5U DNA polymerase (NEB Klenow). Oligonucleotides were denatured at 98°C for 10 min, annealed after cooling slowly to 25°C (1°C/s), and extended at 25°C for at least 2 h. Reactions were heat inactivated at 75°C for 20 min and used directly for Gibson assembly into a digested receiving plasmid with a fixed barcode.

### Cell Culture.

HeLa cells (ATCC) were grown in high-glucose DMEM supplemented with 10% fetal bovine serum and penicillin and streptomycin (ThermoFisher). All cells were grown at 37°C, 5% CO2, and 95% relative humidity.

### Transfection, RNA Extraction, and Reverse Transcription.

Cells were transfected at 60 to 80% confluence with FuGENE HD^®^according to the manufacturer’s protocol at a 3:1 FuGENE HD^®^to DNA ratio. For high-throughput measurements of splicing outcomes, 10 μg pooled reporter assay DNA was transfected in three 100-mm plates. For biochemical analysis of individual reporters, 1 μg or 2.5 μg individual reporter DNA was transfected into each well of a 12- or 6-well plate (respectively). Then, 24 h after transfection, total RNA was isolated from detached cells (Accutase^®^, ThermoFisher). For amplicon sequencing, total RNA was isolated using phenol-chloroform (Ambion) extraction (5PRIME Phase Lock Gel, Quantabio) followed by DNase treatment (TURBO DNase). For biochemical analysis, RNA was isolated using a silica column (illustra™RNAspin Mini RNA Isolation Kit, GE Healthcare) with on-column DNase digestion following the manufacturer’s automated protocol. DNase-treated RNA was reverse transcribed using a reporter-specific primer following the manufacturer’s specifications (SuperScript IV Reverse Transcriptase, Thermo Fisher) with RNase H treatment. Reverse transcription primers included degenerate nucleotides to serve as unique molecular identifiers (UMIs) during amplicon sequencing (46, 47). cDNA products were used for amplicon sequencing or biochemical analysis.

### Amplicon Sequencing.

Amplicon sequencing was used to identify exon–barcode pairings in Lib1 and to quantify splicing products from reverse-transcribed cDNA. Second-strand synthesis added additional UMIs in a single anneal-extend cycle of 98°C for 10 min, cooled slowly to 60°C (0.1°C/s), annealed at 60°C for 5 min, and extended at 72°C for 5 min (Phusion^®^Hot Start Flex 2X Master Mix, NEB). Resulting double-stranded amplicons were amplified using a two-stage procedure. In the first stage, targets were amplified by PCR primers. PCR was performed using the following protocol: 98°C for 30-s initial denaturation, then 16 cycles of 98°C denaturation for 10 s, 60°C annealing for 15 s, 72°C extension for 1 min 45 s, and a final extension step at 72°C for 5 min (Phusion^®^Hot Start Flex 2X Master Mix, NEB). Longer extension times and a minimal number of PCR cycles were used to avoid recombination across exons and barcodes. The number of cycles was determined for each sample by first running 10 μL qPCR reactions (LightCycler^®^480 SYBR Green I Master, Roche). In the second stage, index primers were added using 5 PCR cycles. PCR was performed using the following protocol: 98°C for 30 s initial denaturation, then 5 cycles of 98°C denaturation for 10 s, 71°C annealing for 15 s, 72°C extension for 1 min 45 s, and a final extension step at 72°C for 5 min (Phusion^®^Hot Start Flex 2X Master Mix, NEB). Final DNA concentrations were measured using fluorometric measurements (Qubit 1X dsDNA HS Assay, Thermo Fisher) on a Qubit™ 3 Fluorometer. Paired-end sequencing was carried out on an Illumina NextSeq 550 with 10% PhiX spiked in, with 54 cycles in read 1 (reverse) and 106 in read 2 (forward). About 4M paired-end reads (>10X coverage) were acquired for Lib1 exon–barcode sequencing and an average of 22M paired-end reads (>50X coverage) for each PSI quantification replicate.

### Biochemical Analysis.

PCR amplification reactions to determine splicing products were carried out in 20 μL reactions containing 10 μL OneTaq^®^2X Master Mix with Standard Buffer (NEB), 200 nM each forward and reverse primers (IDT), and 1 μL cDNA. PCR was performed using the following protocol: 94°C for 30-s initial denaturation, then 25 cycles of 94°C denaturation for 10 s, 62°C annealing for 15 s, 68°C extension for 20 s, and a final extension step at 68°C for 1 min. Then, 5 μL final PCR product was run out on 2.0% agarose (Denville Scientific) Tris-acetate-EDTA (TAE) gel with ethidium bromide and visualized on a Bio-Rad imager. Densitometry measurements to calculate PSI were measured using Bio-Rad Image Lab (Windows v6.1).

### Reporter Assay Preprocessing.

The list of all exons in the reporter assay with their corresponding barcodes was extracted from DNA sequencing of Lib1. To ensure unique coupling of barcodes to exons, barcodes appearing with more than one exon sequence were filtered out. This step ignored exon sequences appearing only once, as those are likely due to sequencing errors. Barcodes with fewer than two DNA reads in total were also filtered out.

Next, splicing outcomes were extracted from RNA sequencing of each of the three replicate transfections of Lib2. For each replicate, each read was identified by barcode and was assigned a splicing outcome (exon skipping, exon inclusion, intron retention, splicing inside exon, or unknown splicing). Carryover from Lib1 was filtered out, as were reads for which exon 1 could not be identified. Using unique molecular identifiers (UMIs), the fraction of duplicate reads in each replicate was estimated to be below 23%. The counts from all three replicates were merged for downstream analysis. Barcodes with fewer than 60 total reads, barcodes that contained an Esp3I restriction site in either strand of the exon or its barcode, and barcodes where inclusion and skipping made up less than 80% of all reads were filtered out.

Finally, the dataset was generated by computing PSI for each barcode as

$$\begin{array}{c} \;\text{PSI}=\frac{n_{\text{inclusion}}}{n_{\text{skipping}}+n_{\text{inclusion}}}, \end{array}$$

where $n_{\text{inclusion}}$ is the total number of exon inclusion reads, and $n_{\text{skipping}}$ is the total number of exon skipping reads. In addition to the measured PSI, the dataset includes for each barcode: 1) a 90-nucleotide sequence, containing the 70-nucleotide variable exon sequence plus the 10 fixed flanking nucleotides on each side; 2) structure in dot-bracket notation predicted by RNAFold [Vienna RNA (21), version 2.4.17], using default parameters; 3) an indicator vector indicating which nucleotide participates in a predicted G-U wobble base pair. The dataset was split randomly into a training set and a test set in an 80/20 split, using a fixed seed for reproducibility.

### Model Design.

The model’s input is a triple of vectors $\left(x_{\text{seq}},x_{\text{struct}},x_{\text{wobble}}\right)$,

$$\begin{array}{c} \begin{array}{ccc} & \;\;x_{\text{seq}}\in {\left\{A,C,G,U\right\}}^{d} & \;\;\;[\text{4-category sequence input}]\\ & \;\;x_{\text{struct}}\in {\left\{(\;,\cdot \;,)\right\}}^{d} & \;\;\;[\text{3-category structure input}]\\ & x_{\text{wobble}}\in {\left\{0,1\right\}}^{d}, & \;\;\;[\text{wobble pair indicator input}], \end{array} \end{array}$$

where d=90. The neural network contains four “strength-computation modules” (SCM) defined as

$$\begin{array}{c} f_{a}^{b}:x\mapsto \;\text{Sum}\;\left(\text{Softplus}\;\left(\text{Position-Bias}\;\left(\text{Convolution}\;\left(x;\alpha_{a}^{b}\right);\beta_{a}^{b}\right)\right)\right)\;\left[\;\text{SCM}\right] \end{array}$$

$$\begin{array}{c} \alpha_{a}^{b}\in R^{w_{a}^{b}\times c_{a}^{b}\times k_{a}^{b}},\;\beta_{a}^{b}\in R^{\left(d-w_{a}^{b}+1\right)\times k_{a}^{b}}, \end{array}$$

where a∈{incl,skip}, and b∈{seq,struct}. The input is either $x=[x_{\text{seq}}]$ (sequence SCM) or $x=[x_{\text{seq}},x_{\text{struct}},x_{\text{wobble}}]$ (structure SCM), and the output is a scalar. The 1D convolutional layer contains $k_{\text{a}}^{\text{b}}=20$ convolutional filters of width $w_{\text{a}}^{\text{b}}=6$ for each sequence SCM (b=seq), and $k_{\text{a}}^{\text{b}}=8$ convolutional filters of width $w_{\text{a}}^{\text{b}}=30$ for each structure SCM (b=struct). The number of input channels is $c_{\text{a}}^{\text{b}}=4$ for sequence SCM (corresponding to the one-hot encoded four nucleotides) and $c_{\text{a}}^{\text{b}}=8$ for structure SCM (corresponding to sequence, structure, and wobble indicator). The output of the convolution layer is a $\left(d-w_{\text{a}}^{\text{b}}+1\right)\times k_{\text{a}}^{\text{b}}$ matrix z of “raw” strengths. The position bias layer maps inputs z to $z+\beta_{\text{a}}^{\text{b}}$, adjusting the raw strengths based on position along the exon. Finally, each position-adjusted raw strength is passed through a softplus activation, and the resulting strengths are all summed up to form the output of the SCM $f_{\text{a}}^{\text{b}}$.

The splicing prediction model $m(x_{\text{seq}},x_{\text{struct}},x_{\text{wobble}};\theta )$ is then defined as

[1] $$\begin{array}{cc} & m(x_{\text{seq}},x_{\text{struct}},x_{\text{wobble}};\theta )\\ & \;=\text{Tuner}(f_{\text{incl}}^{\text{seq}}\left(\left[x_{\text{seq}}\right]\right)+f_{\text{incl}}^{\text{struct}}\left(\left[x_{\text{seq}},x_{\text{struct}},x_{\text{wobble}}\right]\right)\\ & \;-f_{\text{skip}}^{\text{seq}}\left(\left[x_{\text{seq}}\right]\right)-f_{\text{skip}}^{\text{struct}}\left(\left[x_{\text{seq}},x_{\text{struct}},x_{\text{wobble}}\right]\right);\gamma ). \end{array}$$

This model computes the total strength for inclusion and for skipping and uses their difference to predict splicing outcomes. The function Tuner(·;γ):R→[0,1] is a learned nonlinear activation function that maps this difference to a PSI prediction. It consists of a 3-layer fully connected network with a residual connection from the input to the output layer, followed by a sigmoid activation. The parameter set θ contains the parameters of all SCMs and the parameter γ.

### Model Training.

The model was implemented in Python 3.8 (48) using Tensorflow 2.6 (49) and Numpy 1.20 (50). Batched gradient descent was used to optimize the model’s parameters using the Adam optimizer, with KL divergence as the loss function. Hyperparameters such as regularization parameters were tuned with grid search. Training the model took about 2 h on a mid-range 4-core with 16 GB of RAM.

To improve interpretability, the model was trained in steps (custom training schedule), progressively adding learnable parameters in each step. First, a simplified model given by

$$\begin{array}{c} \text{Tuner'}(f_{\text{incl}}^{\text{seq}}\left(\left[x_{\text{seq}}\right]\right)-f_{\text{skip}}^{\text{seq}}\left(\left[x_{\text{seq}}\right]\right);\nu ,\eta ) \end{array}$$

was trained. Here, Tuner'(·;ν,η):R→[0,1] is a learned nonlinear activation function defined by x↦σ(νx+η), where σ is the sigmoid function, and ν and η are two real parameters. This step ensures that short sequence motifs are captured by the sequence SCMs and not the more complex structure SCMs. In the second step, the structure SCMs were added, leading to a model identical to the final one Eq. **1**, except for the use of Tuner' instead of Tuner. The sequence SCM weights were initialized to those of the previous model. In the third and last step, the Tuner function was introduced, leading to the final model Eq. **1**. SCM weights were initialized to those of the previous model.

To further improve the model’s interpretability, regularization terms were added. First, to obtain a concise list of filters, an activity regularization loss term was used. The term consists of the $\ell_{1}$ norm of all the strengths. Second, a smoothness regularization loss term was applied to position bias layer weights. This term consists of the $\ell_{2}$ norm of the discrete derivative of the weight vectors (defined as the difference between the vector and itself shifted by one along the sequence dimension). Each of the two loss terms was multiplied by a hyperparameter.

Hyperparameters were optimized based on two criteria: held-out KL divergence and sparsity of activations. Sparsity was measured as the minimum number of activations needed per exon to achieve KL below a threshold. Among all hyperparameters leading to sufficiently high accuracy and sparsity, the one with the highest smoothness regularization was chosen.

### Prediction Accuracy on Other Assays.

Exon sequences and PSI measurements were obtained from previous publications. Exons including indel mutations or differing from WT sequence in the first or last three nucleotides were filtered out. In order to generalize the model to exons of varying lengths, we applied Lanczos resampling (with parameter a=3) to the position bias weights. To account for differences in splice sites, flanking sequences, and cell types, one scalar correction term was introduced per assay, effectively adjusting the basal strength (B) (17).

### Filter Visualization.

To avoid reporting redundant sequence filters, hierarchical clustering using SciPy (51) was applied. Each sequence filter was represented by a vector containing its total strength for each of the exons in the dataset. The strongest filter in each cluster was then used to generate a sequence logo (25). The logo represents the set of 6-mers that lead to positive filter activation.

The structure filters included one G-poor filter and three stem loop filters. Since enumerating all 30-mers is not tractable, the G-poor sequence logo was computed by evaluating the filter on a subset of sequences from our dataset. As the three stem loop filters differed in the length of the loop (short, medium, long) but were otherwise very similar, they were considered as one cluster. Layer-wise relevance propagation was used to visualize individual nucleotide contributions to filter strength (52).

### Ruling Out Sequencing Artifacts.

Our model identified two uncharacterized exon-skipping features (stem loop structures and G-poor sequences). As our dataset was generated using multiple enzymatic reactions (from reverse transcription to polymerase amplification), we were concerned that rather than learning novel splicing features, our model instead learned artifacts introduced during data generation. Indeed, previous work noted that both structured and unstructured nucleic acid regions can impair enzymatic reactivity (53). If these features impaired enzymatic reactivity, we would expect to observe distinct biases in the absolute number of sequencing reads for such exons. Specifically, for exons containing stem loop structures or G-poor sequences, we would expect an undercounting of inclusion reads $n_{\text{inclusion}}$ (due to the inability to sequence reads containing the feature in the included exon) and, crucially, no change in the number of skipping reads $n_{\text{skipping}}$ (since such reads do not contain the exon). In contrast, a bona fide exon skipping feature should appear as a reduction in $n_{\text{inclusion}}$ accompanied by an increase in $n_{\text{skipping}}$. An analysis of our sequencing data supports that the stem loops and G-poor sequences are bona fide exon-skipping features rather than sequencing artifacts (*SI Appendix*, Fig. S9).

### Design of Mutant Constructs.

To validate the stem loop feature, candidate exons with high medium-length stem loop filter strength (top percentile) but with no other stem loop activations elsewhere in the exon were selected. Three mutants of each such exon were then generated. To ensure these mutants do not introduce or disrupt other features, exons where this mutation significantly changed strengths of other filters were filtered out.

To validate the G-poor stretch feature, candidate exons that strongly activate the G-poor filter exactly once along the exon were selected. For each candidate exon, a C-to-G mutation in the middle of the activated filter’s window was introduced. As before, to ensure this does not introduce or disrupt other features, exons where this mutation significantly changed strengths of other filters were filtered out.

## Supplementary Material

Appendix 01 (PDF)

Dataset S01 (CSV)

Dataset S02 (CSV)

Dataset S03 (XLSX)

Dataset S04 (XLSX)

## Data Availability

Sequence data that support the findings of this study have been deposited in the Gene Expression Omnibus under accession number GSE200096 (54). Custom code, preprocessed datasets, and trained model are available on GitHub (https://github.com/regev-lab/interpretable-splicing-model) (55). Plasmids used in this study will be available through AddGene.
