# Supplementary material for: Deciphering RNA splicing logic with interpretable machine learning
Source: Proc Natl Acad Sci U S A. 2023 Oct 5;120(41):e2221165120. doi: 10.1073/pnas.2221165120 (PMC10576025; doi:10.1073/pnas.2221165120)
Supplement: Supplementary file 1 — Appendix 01 (PDF) [file pnas.2221165120.sapp.pdf]

Supporting Information for

Deciphering RNA splicing logic with interpretable machine learning

Susan E. Liao, Mukund Sudarshan, and Oded Regev

Oded Regev
E-mail: regev@cims.nyu.edu

This PDF file includes:

Figs. S1 to S9
Table S1
Legends for Dataset S1 to S4
SI References

Other supporting materials for this manuscript include the following:

Datasets S1 to S4

Table S1. Predictive accuracy of interpretable model on gene-derived reporter assays.

Exon	Publication	Datapoints	Exon Length (nt)	Cell Line	Prediction accuracy (R^2)
Random sequences	Our dataset	$> 3 \times 10^5$	76	HeLa	0.822
WT1 exon 5	(1)	5560	51	HEK293	0.533
FAS exon 6	(2)	794	63	HEK293	0.845
SMN2 exon 7	(3–5)	56	54	C33A	0.337
SMN1 exon 7	(3–5)	40	54	HEK293	0.734
BRCA2 exon 7	(7)	31	115	HEK293	0.686
CFTR exon 13	(8)	22	87	HEK293	0.686

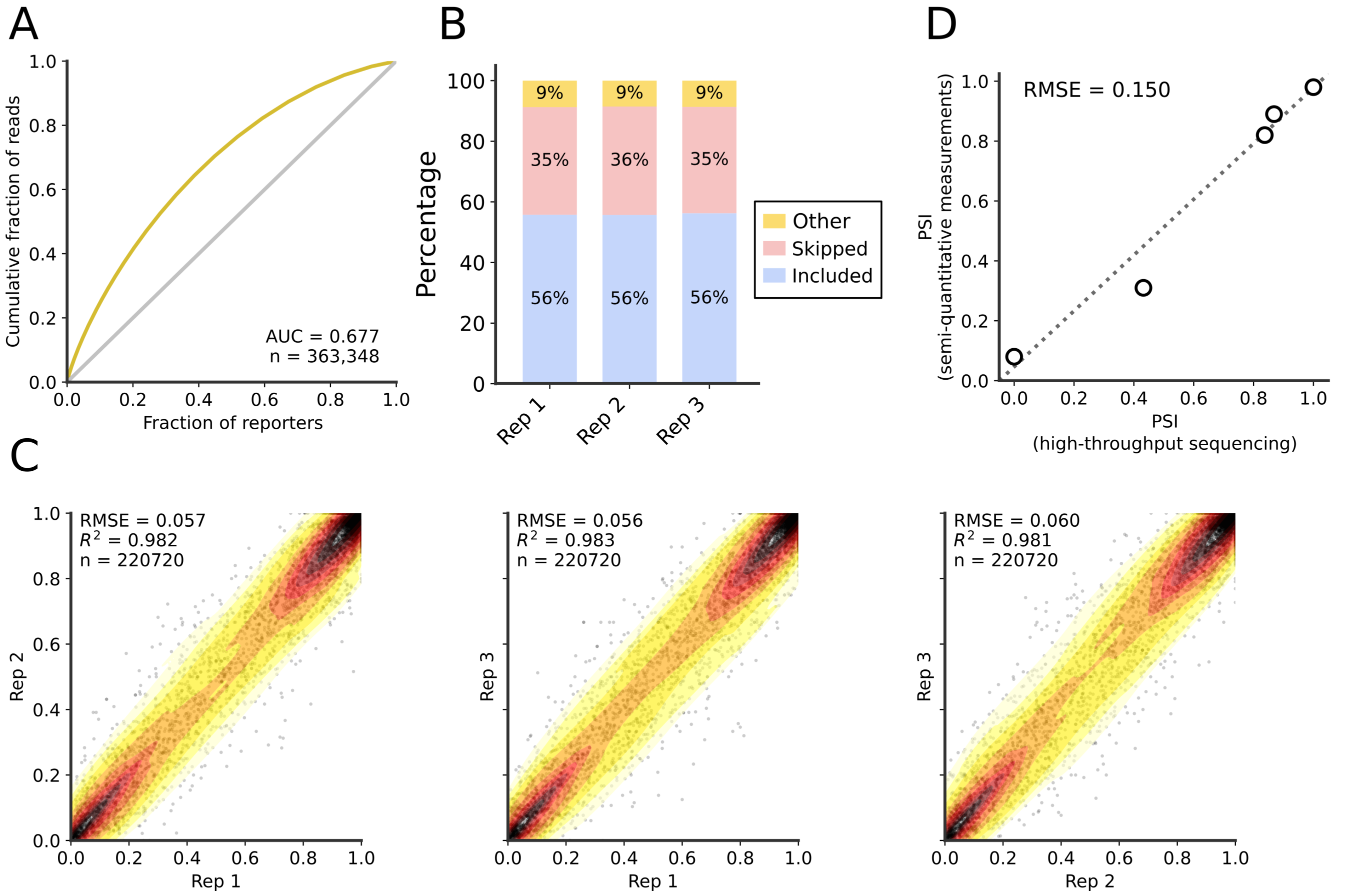

Fig. S1. Assay quality checks. (A) Lorenz plot showing the distribution of reads across the reporters in a high-throughput sequencing of the library DNA (gold). A perfectly even library would be on the diagonal (gray). **(B)** Over 90% of splicing products corresponded to exon inclusion or exon skipping products, as measured by high-throughput RNA sequencing. **(C)** Comparison of PSI measurements across the three biological replicates. **(D)** Comparison of PSI measurements from high-throughput sequencing and semi-quantitative measurements for five individual reporters (V1-V5). AUC: area under curve; RMSE: root-mean-square error; R^2: coefficient of determination.

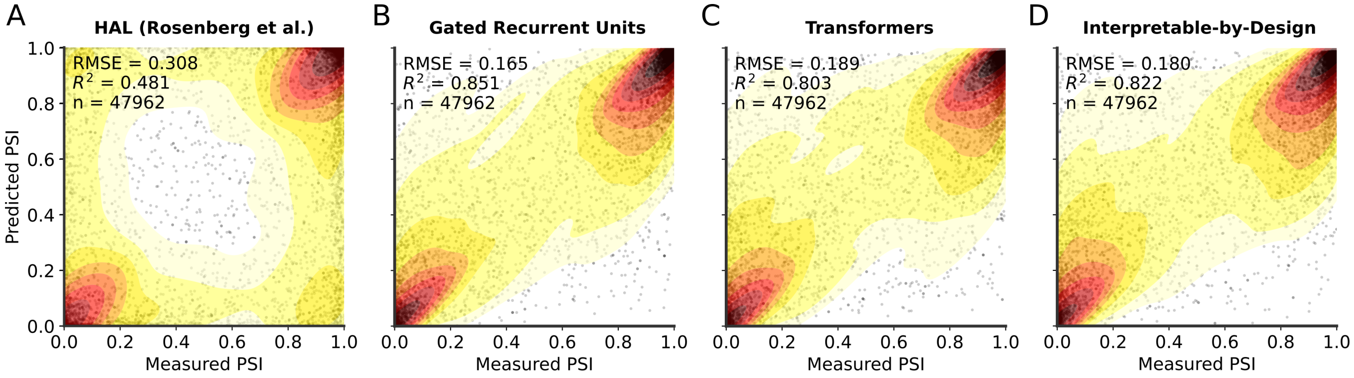

Fig. S2. Model predictive accuracy. Predictions on the held-out experimental data of HAL, state-of-the-art models (gated recurrent units and transformers) and the interpretable-by-design model.

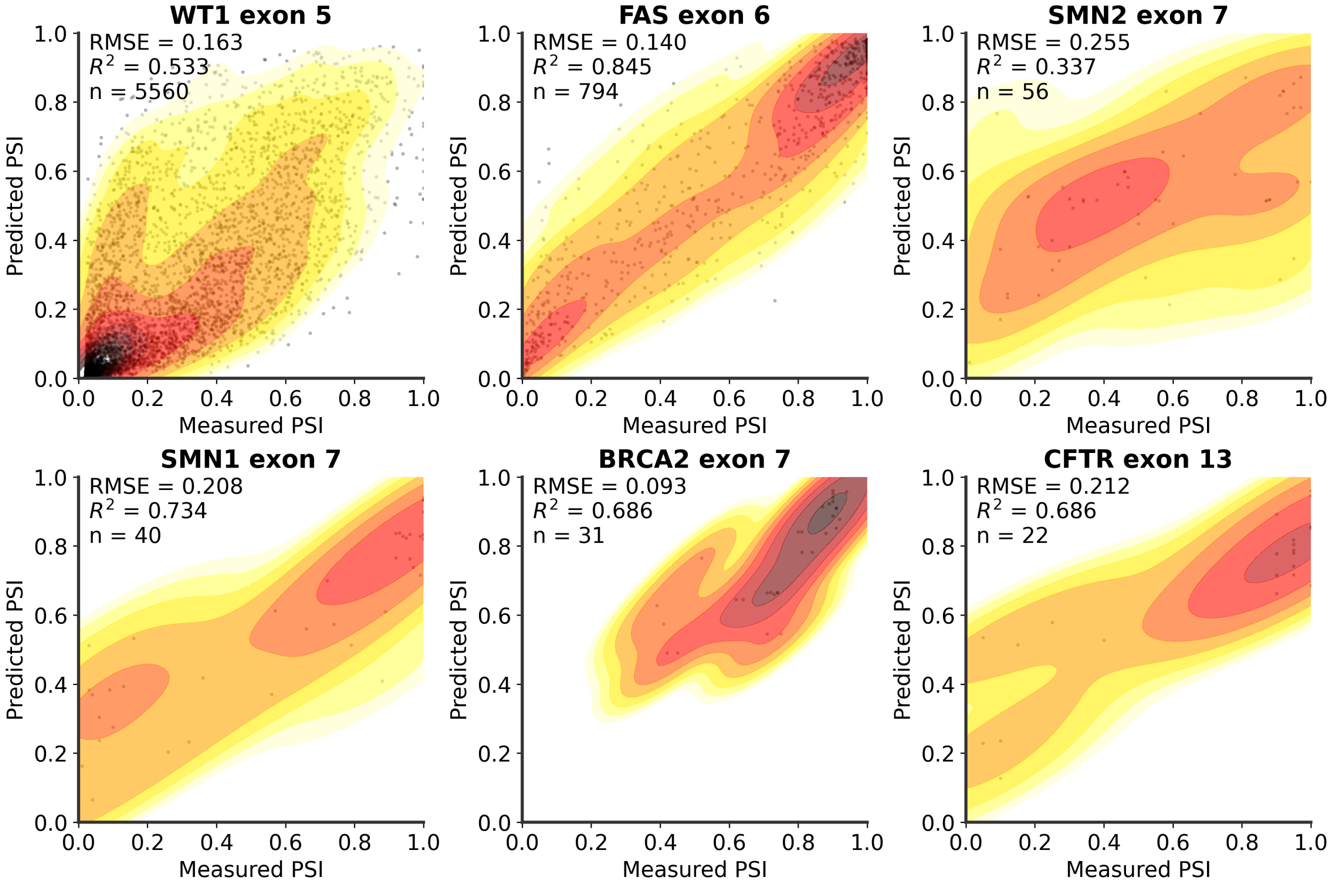

Fig. S3. Predictive accuracy of interpretable-by-design model on other datasets. Previously-published datasets include: WT1 exon 5 (HEK293 cells) (1), FAS exon 6 (HEK293 cells) (2), SMN2 exon 7 and SMN1 exon 7 (C33a cells) (3–6), BRCA2 exon 7 (7), and CFTR exon 13 (8).

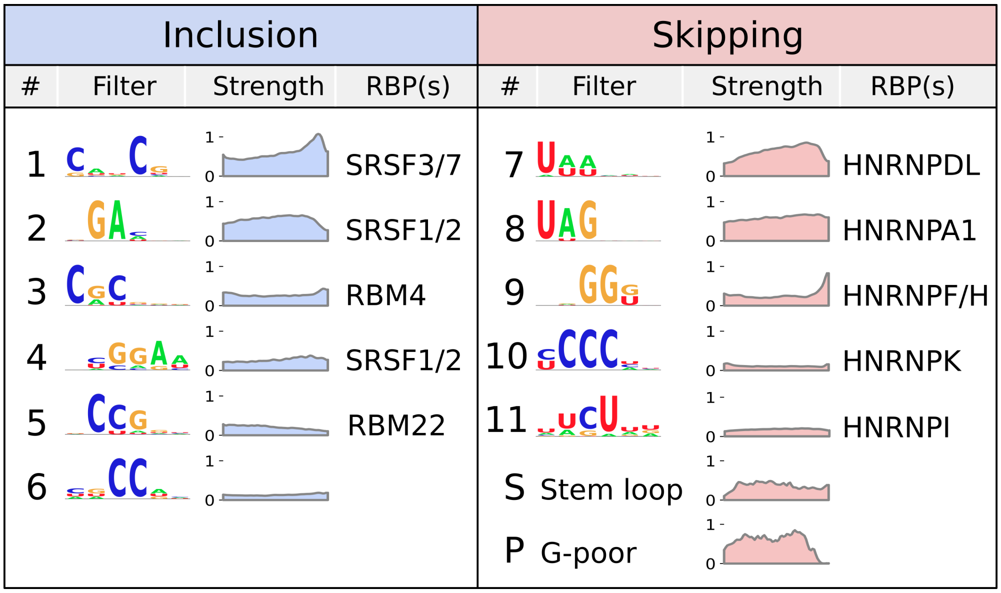

Fig. S4. Model expands on known splicing logic (related to Figure 2). Splicing features detected by the model's filters, represented by their sequence logo (9). Filters either contribute to inclusion (blue) or skipping (red). Plots show the average strength in our dataset of each filter as a function of position along the exon. RNA binding proteins (RBP) with a similar binding motif, as reported in previous work (10–12). The model also identified short stem loops and long G-poor stretches as contributing to exon skipping.

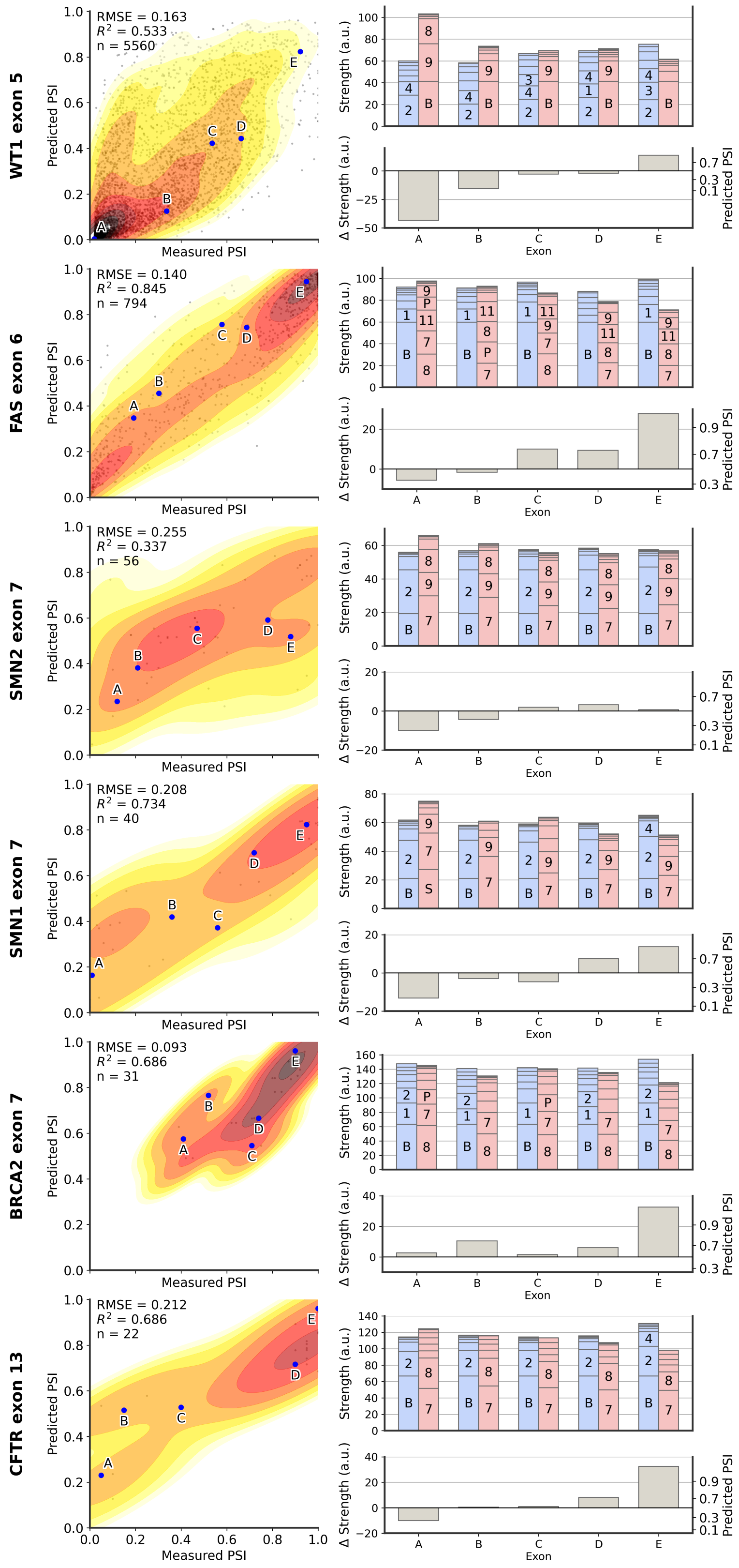

Fig. S5. Balance plots of interpretable-by-design model on other datasets. Left: Predictions of the interpretable-by-design model on previously-published assays, as in Fig. S3. Right: balance plots showing the strengths of activated filters leading to the predicted PSI for each of the five representative exons highlighted on the left.

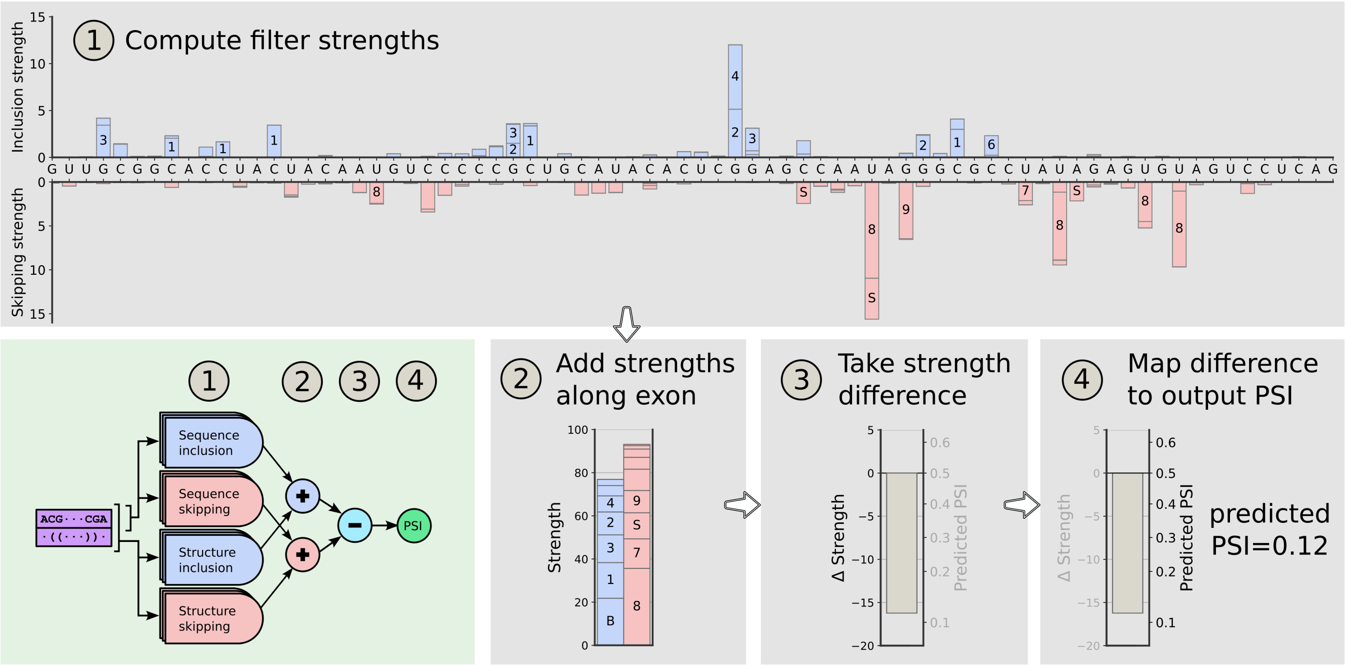

Fig. S6. Tracing the neural network computation from exon sequence to predicted PSI. Our neural network (bottom left) arrives at its predictions in four steps. First, using exon sequence and predicted structure, it computes filter strengths for each position along the exon (1). Next, it adds all inclusion strengths together and all skipping strengths together (2). Then, the difference between these two strengths (Δ strength) is computed (3). Finally, it maps that difference to a predicted output PSI (4).

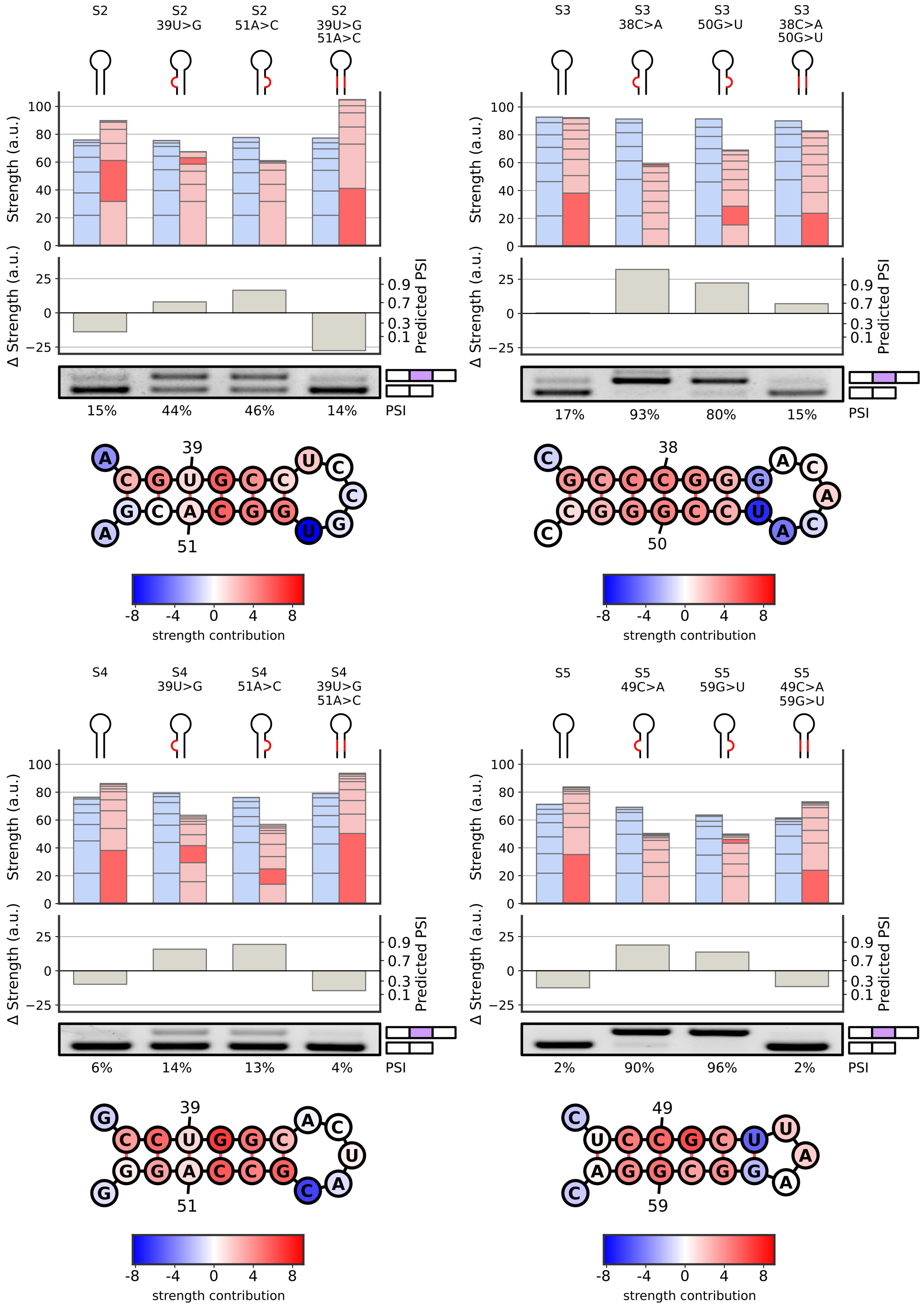

Fig. S7. Additional validation of novel stem loop feature. Four more exons (S2, S3, S4, S5) were chosen and validated as in Fig. 4.

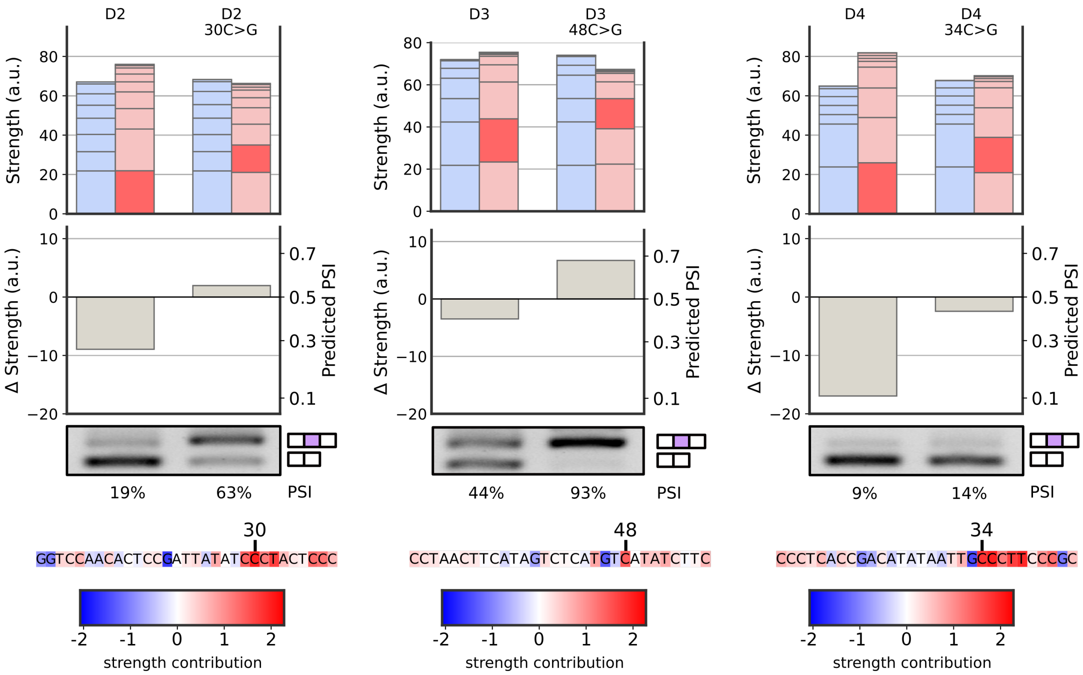

Fig. S8. Additional validations of the G-poor feature. Three more exons (D2, D3, D4) were chosen and validated as in Fig. 5.

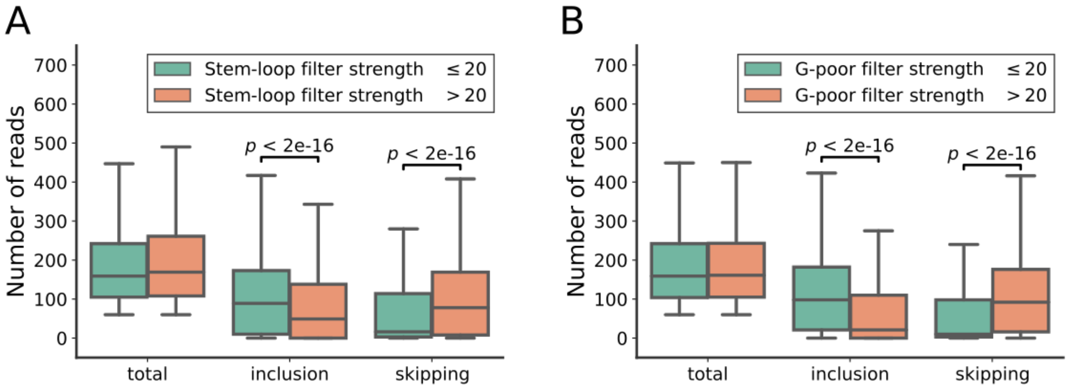

Fig. S9. Effect of novel features on absolute read counts. (A) Box plot showing the distribution of the total number of sequencing reads, the number of exon inclusion reads, and the number of exon skipping reads, for exons with stem loop strength at most 20 and greater than 20. **(B)** As in panel (A) for G-poor strengths. Center line: median; box limits: upper and lower quartiles; whiskers: 1.5x interquartile range. p values: Student's t-test.

SI Dataset S1 (training_data.csv)
All exons in the training dataset with their measured PSI.

SI Dataset S2 (test_data.csv)
All exons in the held-out test dataset with their measured and predicted PSI.

SI Dataset S3 (supplementary_1.xlsx)
All validation constructs with their predicted PSI.

SI Dataset S4 (supplementary2_primers.xlsx)
List of primers.

References

1. S Ke, et al., Saturation mutagenesis reveals manifold determinants of exon definition. *Genome research* **28**, 11–24 (2018).
2. P Baeza-Centurion, B Miñana, JM Schmiedel, J Valcárcel, B Lehner, Combinatorial genetics reveals a scaling law for the effects of mutations on splicing. *Cell* **176**, 549–563.e23 (2019).
3. NN Singh, EJ Androphy, RN Singh, An extended inhibitory context causes skipping of exon 7 of SMN2 in spinal muscular atrophy. *Biochem. biophysical research communications* **315**, 381–8 (2004).
4. NN Singh, EJ Androphy, RN Singh, In vivo selection reveals combinatorial controls that define a critical exon in the spinal muscular atrophy genes. *RNA (New York, N.Y.)* **10**, 1291–305 (2004).
5. NN Singh, RN Singh, EJ Androphy, Modulating role of RNA structure in alternative splicing of a critical exon in the spinal muscular atrophy genes. *Nucleic acids research* **35**, 371–89 (2007).
6. AB Rosenberg, RP Patwardhan, J Shendure, G Seelig, Learning the sequence determinants of alternative splicing from millions of random sequences. *Cell* **163**, 698–711 (2015).
7. D Di Giacomo, et al., Functional analysis of a large set of brca2 exon 7 variants highlights the predictive value of hexamer scores in detecting alterations of exonic splicing regulatory elements. *Hum. mutation* **34**, 1547–57 (2013).
8. F Pagani, M Raponi, FE Baralle, Synonymous mutations in cftr exon 12 affect splicing and are not neutral in evolution. *Proc. Natl. Acad. Sci. United States Am.* **102**, 6368–72 (2005).
9. TD Schneider, RM Stephens, Sequence logos: a new way to display consensus sequences. *Nucleic acids research* **18**, 6097–100 (1990).
10. Y Cavaloc, CF Bourgeois, L Kister, J Stévenin, The splicing factors 9G8 and SRp20 transactivate splicing through different and specific enhancers. *RNA (New York, N.Y.)* **5**, 468–83 (1999).
11. D Ray, et al., A compendium of RNA-binding motifs for decoding gene regulation. *Nature* **499**, 172–7 (2013).
12. D Dominguez, et al., Sequence, structure, and context preferences of human RNA binding proteins. *Mol. Cell* **70**, 854–867.e9 (2018).
